# Evaluation of Nitrogen Nutrition in Diminishing Water Deficiency at Different Growth Stages of Maize by Chlorophyll Fluorescence Parameters

**DOI:** 10.3390/plants9060676

**Published:** 2020-05-27

**Authors:** Attila Simkó, Gáspár Soma Gáspár, László Kiss, Péter Makleit, Szilvia Veres

**Affiliations:** Department of Agricultural Botany, Crop Physiology and Biotechnology, Institute of Crop Sciences, University of Debrecen, H-4032 Debrecen, Hungary; simko.attila@agr.unideb.hu (A.S.); gasparsomagaspar@gmail.com (G.S.G.); laszlo.kiss02@gmail.com (L.K.); pmakleit@agr.unideb.hu (P.M.)

**Keywords:** *Zea mays*, potential photochemical efficiency, yield, nitrogen, drought

## Abstract

Efficient nitrogen (N) nutrition has been reported to have the potential to alleviate the drought stress damages by maintaining metabolic activities even at low tissue water potential. The goal of our research was to find a correlation on the genotype level between the effect of different amounts of nitrogen nutrition and water supply at different growth stages. A small-plot experiment was established with three maize hybrids and three levels of nitrogen, and two different amounts of water supply were applied during the vegetation period of 2018 and 2019. Chlorophyll fluorescence parameters were detected, as well as potential and actual photochemical efficiency of PSII, at three growth stages: eight-leaf stage, tasseling, silking. At physiological maturity, the yield of hybrids was also measured. While only genotype differences were described among the investigated parameters in the V8 stage, treatment effects were also realized based on the measured chlorophyll fluorescence parameters during the tasseling and silking stages. Beyond the significant effect of irrigation, a similar impact was declared in the case of 80 kg ha^−1^ N treatment at the later growth stages. Pronounced correlation was described between chlorophyll fluorescence parameters and yield mainly under irrigated conditions. Our result suggested that lower N nutrition may be sufficient mainly under irrigated conditions, and in vivo chlorophyll fluorescence parameters are appropriate for detecting the effect of environmental factors in different growth stages.

## 1. Introduction

Water is an essential element for living organisms. Water deficiency negatively affects plant growth mainly through the nutrient availability in the soil. In recent years, the amount of annual rainfall has varied considerably all over the world and in Hungary as well. Increasing temperature trends affecting crop production throughout Europe furthermore increase the frequency of drought crop years and negatively affect agriculture in Southern and Central Europe [1]. Generally, the frequency of extreme rainfall and extreme drought is increasing. In the future, our country’s climate will become drier, according to Mika’s predictions [2]; Zampieri [3] projects a 2 °C increase in the global average temperature.

Nitrogen (N) is one of the most important nutrients in crop production, but the application of this element often exceeds crop demands. N use efficiency of genotypes is different [4,5]. A decrease in N use efficiency was observed at higher rates of N fertilizers [6]. One of the most significant effects of irrigation can be observed during nutrient management. It has been found that under non-irrigated conditions, a dose of 90 kg ha^−1^ of N with the corresponding dose of phosphorus and potassium is optimal, whereas under irrigated conditions, a dose of 120 kg ha^−1^ is considered appropriate [7]. Several studies have proven the correlation among N supply and relative chlorophyll meter readings also known as Soil Plant Analysis Development (SPAD) values [8,9,10]. Nagy [11] declared that fertilization had a significant effect on the SPAD value under both irrigated and non-irrigated conditions, but the value increased to a higher rate with irrigated conditions. The effect of irrigation was significant in all cases compared to the non-irrigated areas. According to Kamara’s [12] results, drought tolerance of maize genotypes can relate to tolerance towards N deficiency. These genotypes accumulate more N and had more efficient N uptake.

Maize (*Zea mays* L.) belongs to the group of the most important crops in Hungary and worldwide. In recent decades, the average yield of maize has increased [13]. Nevertheless, yield loss of maize production from drought is expected as a result of rising temperatures and changes in rainfall distribution [14], although the non-linear response of yield loss risk highlighted the increase in drought severity [15]. Plant breeding programs and technological innovations, like tools of precision agriculture, can reduce the effect of these problems, but we need to carefully consider the local environmental conditions [16,17]. Irrigation is a relatively expensive option to reduce the negative effects of water deficiency on plants, and if we integrate irrigation into the crop growing system, we need to modify the agrotechnical elements (tillage system, nutrient supply, etc.).

Studying the phenomenon of chlorophyll fluorescence is a widespread method in plant stress physiology research [18,19,20]. The effect of drought depends on its severity. Mild drought does not affect the efficiency of photosystem two (PSII) but stomatal closure can decrease the CO_2_ assimilation; however, a higher level of drought negatively affects the adenosine triphosphate (ATP) metabolism [21]. Dias and Bürgemann [22] observed that chlorophyll fluorescence quenching, photosystem II quantum yield, and electron transport rate were decreased due to drought. Based on Faraloni’s [23] results, potential photochemical efficiency of PSII (Fv/Fm) is suitable for drought stress research. Some results claimed that water supply does not have any effect on the potential photochemical efficiency of photosystem II and apparent photosynthetic electron transport rate [24]. Li [25] also reported severe drought caused a decrease in chlorophyll content, the optimal efficiency of PSII photochemistry (Fv/Fm), and photochemical quenching in maize. Gholamin and Khayatnezhad claimed the amount of minimal fluorescence (Fo) was increased, while the chlorophyll content and Fv/Fm ratio were reduced for the effect of drought in maize [26]. Liu [27] reported the decrease of the Fv/Fm ratio for the effect of drought stress but did not report an experienced difference between genotypes in the case of this parameter. Our main goal was to prove the applicability of a chlorophyll fluorescence induction method for drought stress research and yield estimation on a genotype level and different growth stages. Furthermore, we tested the hypothesis that N fertilization is able to diminish water deficiency in different maize genotypes with different phenology.

## 2. Results

### 2.1. Weather Conditions

**Figure 1:**
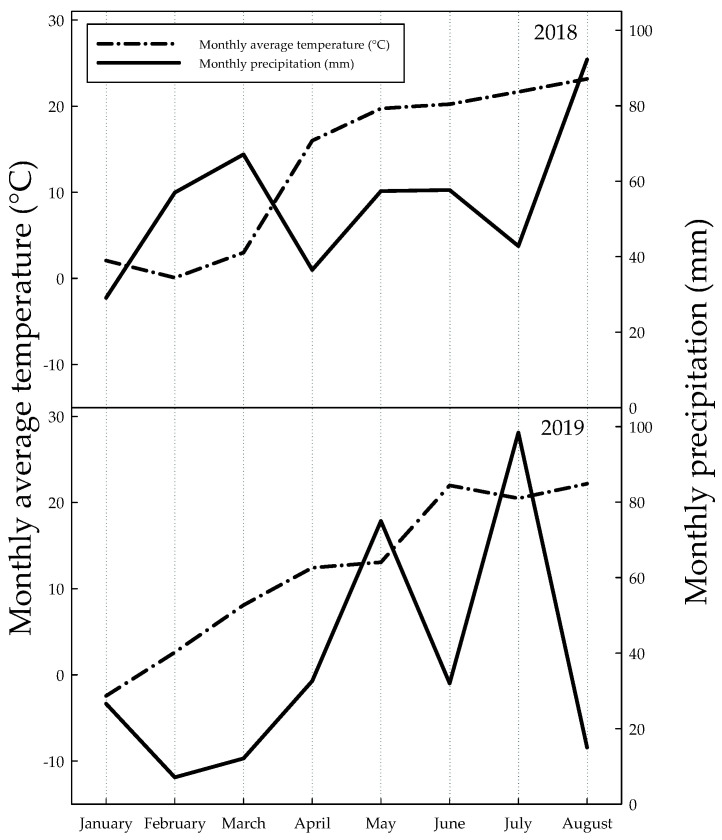
Changes of monthly average temperature (°C) and monthly precipitation (mm) from January 1 to August 31 in 2018 and 2019.

During the experiment, the most important weather data were recorded at the meteorological station near the experimental site. According to the results, in 2018, more precipitation fell than in 2019 during the examined period (Figure 1).

Nevertheless, the weather conditions were favorable in 2019 for maize production. This can be explained by the distribution of precipitation. In May, June, and July of 2018, 157.9 mm rain fell. In contrast, during the same period in 2019, 205.4 mm was the amount of rainfall. The mentioned period is critical from the viewpoint of maize production. To characterize the severity of drought the Gaussen–Banouls xerothermal index was used (Table 1).

Based on the index, only July can be classified as a drought month—when the value is lower than 1—in 2018. However, in 2019, March, June, and August were drought months from the ecological point of view.

### 2.2. Eight-Leaf Stage (V8)

At the V8 phenological stage, a significant difference could only be detected in Fm values in 2018 (Table 2). At this time, significant differences were found between genotypes, but the fertilizer level had no effect on any parameter. Armagnac (1.47 ± 0.02) had a remarkably higher Fm value than Loupiac (1.37 ± 0.03). In 2019, significant differences were found between genotypes but in more parameters than in 2018. Armagnac has significantly higher Fo (0.27 ± 0.003), Fm (1.35 ± 0.02), and Fv (1.08 ± 0.02) values than Loupiac’s Fo (0.26 ± 0.004), Fm (1.26 ± 0.02), and Fv (1.00 ± 0.02), and the Fm value was significantly higher in Fornad (1.32 ± 0.03) compared to Loupiac. A statistically justifiable difference was not observed in any other parameter at the eight-leaf stage (Table 2).

### 2.3. Tasseling Stage (VT)

**Figure 2:**
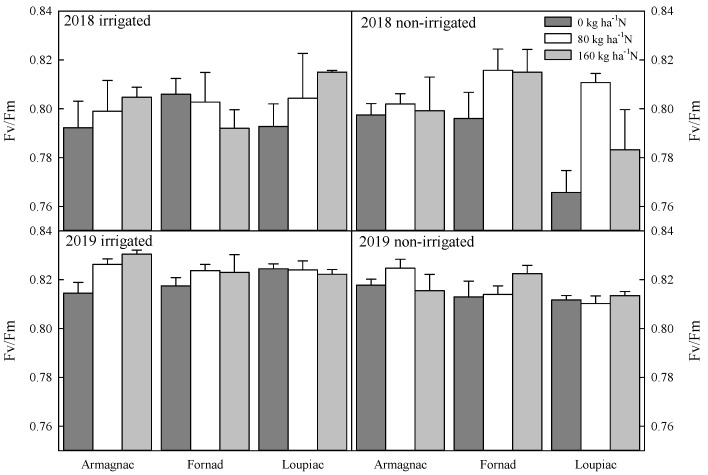
Changes of the maximum quantum yield of photosystem II (Fv/Fm); the effect of maize hybrids (Armagnac, Loupiac, Fornad), different N supply (0 kg ha^−1^; 80 kg ha^−1^; 160 kg ha^−1^), and two irrigation varieties (irrigated, non-irrigated) in a two year experiment (2018, 2019) at the tasseling (VT) phenological stage *n* = 4, ± s.e (differences between means of years (2018, 2019) were significant (*p* ≤ 0.001): in 2019, significantly higher values were observed than in 2018).

At the VT phenological stage, significant differences were observed in every examined parameter, but the effect of the treatments and genotypes were also different in 2018 and 2019. In 2018, a difference was observed between Armagnac (0.27 ± 0.004) and Loupiac (0.26 ± 0.003) in the parameter of minimal fluorescence yield (Fo). In maximum fluorescence yield (Fm), a significant difference was noticed between Armagnac (1.32 ± 0.03) and Loupiac (1.23 ± 0.03), as well as between Fornad (1.32 ± 0.03) and Loupiac. Furthermore, there is a statistically significant interaction between fertilizer, irrigation, and genotype. By examining the variable fluorescence (Fv) parameter, similar results were found. A significant difference was noticed between Armagnac (1.06 ± 0.02) and Loupiac (1.06 ± 0.03), as well as between Fornad (0.98 ± 0.03) and Loupiac. Furthermore, there is a statistically significant interaction between fertilizer, irrigation, and genotype. Irrigation has a significant effect on actual photochemical efficiency (yield). Under irrigated condition, the value of the parameter was 0.44 ± 0.03, while the mean of non-irrigated plots was 0.36 ± 0.04. Remarkable differences were found in case of maximum quantum yield of photosystem II (Fv/Fm) in 2018 (Figure 2).

**Figure 3:**
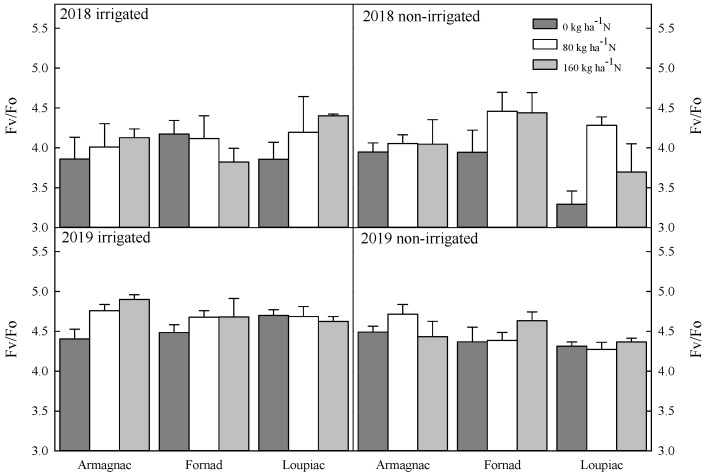
Changes in the ratio between variable and minimal fluorescence (Fv/Fo), the effect of maize hybrids (Armagnac, Loupiac, Fornad), different N supply (0 kg ha^−1^; 80 kg ha^−1^; 160 kg ha^−1^), and two irrigation varieties (irrigated, non-irrigated) in a two year experiment (2018, 2019) at the tasseling (VT) phenological stage *n* = 4, ± s.e. (differences between means of years (2018, 2019) were significant (*p* ≤ 0.001): in 2019, significantly higher values were observed than in 2018).

The 80 kg ha^−1^ level of N has an effect on this parameter. The mentioned level of N was observed as 0.85 ± 0.004, and it was significantly higher than the value at 0 kg ha^−1^ level (0.79 ± 0.004). There is no significant interaction between any factors. The observation was similar in the case of the Fv/Fo ratio (Figure 3).

**Figure 4:**
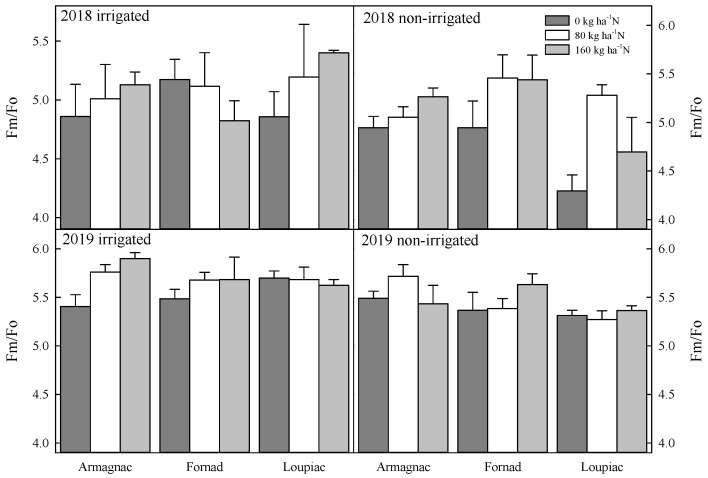
Changes of the ratio between maximal and minimal fluorescence (Fm/Fo), the effect of maize hybrids (Armagnac, Loupiac, Fornad), different N supply (0 kg ha^−1^; 80 kg ha^−1^; 160 kg ha^−1^), and two irrigation varieties (irrigated, non-irrigated) in a two year experiment (2018, 2019) at the tasseling (VT) phenological stage *n* = 4, ± s.e. (differences between means of years (2018, 2019) were significant (*p* ≤ 0.001): in 2019, significantly higher values were observed than in 2018).

A significantly higher Fv/Fo value was experienced in 80 kg ha^−1^ (4.19 ± 0.10) than 0 kg ha^−1^ (3.85 ± 0.10) in 2018. In the case of the Fm/Fo ratio, a similar trend was remarked (Figure 4). At the level of 80 kg ha^−1^ fertilizer (5.19 ± 0.10), essentially higher values were noticed than at a level of 0 kg ha^−1^ (4.85 ± 0.10) N. In 2019, in minimum fluorescence yield (Fo), a significant difference was noticed between Armagnac (0.23 ± 0.003) and Loupiac (0.25 ± 0.004), as well as between Fornad (0.23 ± 0.003) and Loupiac. By examining the maximum fluorescence yield (Fm) values, similar results were found between Armagnac (1.30 ± 0.03) and Loupiac (1.35 ± 0.03). In variable fluorescence (Fv), a significant difference was noticed between Armagnac (1.07 ± 0.02) and Loupiac (1.10 ± 0.02). Additional irrigation also had an effect on this parameter. In the irrigated plots (1.09 ± 0.02) remarkably higher values were noticed than in non-irrigated plots (1.04 ± 0.02).

Remarkable differences were found in the case of maximum quantum yield of photosystem II (Fv/Fm) in 2019 (Figure 2). Among 160 kg ha^−1^ (0.82 ± 0.002) and 0 kg ha^−1^ (0.81 ± 0.002) levels of N, a significant difference was found in Fv/Fm values. Irrigation also had remarkable effect on this parameter. The value was 0.82 ± 0.001 in irrigated and 0.81 ± 0.002 in non-irrigated plots. The observation was similar in the case of the Fv/Fo ratio (Figure 3). A significantly higher Fv/Fo value was experienced in 160 kg ha^−1^ (4.43 ± 0.09) than 0 kg ha^−1^ (4.46 ± 0.05) in 2019. In the case of the Fm/Fo ratio, a similar trend was remarked (Figure 4). At the level of 160 kg ha^−1^ fertilizer (5.61 ± 0.06), essentially higher values were noticed than at the level of 0 kg ha^−1^ (5.46 ± 0.05) N. A strongly significant difference was found among irrigated and non-irrigated plots in both the Fv/Fo and the Fm/Fo ratio.

### 2.4. Silking Stage (R1)

At the third measuring time, significant differences were evinced in just a few cases. In 2018, a statistically proven difference was found in the case of Fm and Fv values (Table 3).

Additional water supply affected these values (Table 2). Surprisingly, higher values were observed in the non-irrigated plots than the treated ones. The difference of means was 0.064 in Fm and 0.059 in Fv. In 2018, statistically certified interaction was observed among irrigation and the fertilizer level, but just in the case of Fo. The effect of irrigation could be observed only in plots treated with 0 kg ha^−1^. Furthermore, the effect of fertilizers could be observed only in non-irrigated plots. In 2019, statistically significant interaction was found between fertilizer and irrigation in Fv/Fm, Fv/Fo, and Fm/Fo parameters at the third measurement (Table 2). The effect of fertilizer could be observed within non-irrigated plots. Without irrigation, the 80 kg ha^−1^ level of N induced remarkably higher values than the 0 kg ha^−1^ 160 kg ha^−1^ level of N in the case of Fv/Fm, Fv/Fo, and Fm/Fo parameters (Table 4). In Fv/Fm, the difference between the 80 kg ha^−1^ and the 160 kg ha^−1^ N supply was 0.03 and 0.02 between the 0 kg ha^−1^ and the 80 kg ha^−1^ portion. In Fv/Fo, the difference between the 80 kg ha^−1^ and the 160 kg ha^−1^ N supply was 0.62 and 0.51 between the 0 kg ha^−1^ and the 80 kg ha^−1^ portion.

In Fm/Fo, the difference between the 80 kg ha^−1^ and the 160 kg ha^−1^ N supply was 0.62 and 0.51 between the 0 kg ha^−1^ and the 80 kg ha^−1^ portion. A statistically significant difference between irrigated and non-irrigated values could be noticed only at the 160 kg ha^−1^ level of N supply in the Fv/Fm, Fv/Fo, and Fm/Fo (Table 4) parameters.

### 2.5. Physiological Maturity

Results of physiological maturity are presented in Table 5. In the case of ear weight, differences were observed between applied N levels in 2018. At 80 kg ha^−1^ (213.07 g ± 5.74) and 160 kg ha^−1^ (206.92 g ± 5.07) of N, significantly higher values were observed than at 0 kg ha^−1^ (176.09 g ± 9.17).

A difference was not evinced between higher levels of N. In 2019, at 80 kg ha^−1^ (185.19 g ± 6.46) and 160 kg ha^−1^ (204.57 g ± 5.40) levels of N, significantly higher values were observed than at 0 kg ha^−1^ (159.82 g ± 8.37). A difference was not evidenced at higher levels of N. Irrigation positively affected this parameter in 2019. The difference of means of irrigated and non-irrigated plots was 16.35 g. Kernel weight per ear was also affected by fertilizer level in 2018 and 2019. In 2018, remarkably higher values were noticed at 160 (184.48 g ± 4.57) and 80 kg ha^−1^ (191.22 g ± 5.13) than at 0 kg ha^−1^ (156.46 g ± 8.19). In 2019, a significantly higher kernel weight was observed at 80 kg ha^−1^ (165.21 g ± 5.91) than at 0 kg ha^−1^ (141.52 g ± 7.38); furthermore, 160 kg ha^−1^ (182.78 g ± 4.78) caused a powerful deviation compared to the other levels of N. The average kernel weight per ear of irrigated plots (170.37 g ± 5.58) was remarkably higher than that of the non-irrigated plots (155.96 g ± 5.60). The kernel/cob ratio was not affected by irrigation in the examined years. In 2018, only 80 kg ha^−1^ (8.90 ± 0.24) had an effect on the kernel/cob ratio, but in 2019, just a 160 kg ha^−1^ (8.51 ± 0.21) dose of N had an effect on the parameter. Remarkable differences were observed between genotypes in 2018 and 2019 as well (Table 6). In the first year of the experiment, the highest values were observed in the case of Fornad (9.67 ± 0.18), which was higher than Loupiac by 1.33 and higher than Armagnac by 2.11. In 2019, a statistically significant difference was found between Fornad (8.88 ± 0.24) and Armagnac (7.36 ± 0.18) and, furthermore, between Loupiac (8.53 ± 0.16) and Armagnac. An interaction was not evinced between treatments.

Pearson’s correlation tests were performed between ear weight, kernel weight per ear, and the Fv/Fm, Fv/Fo, and Fm/Fo parameters obtained from the VT period (Table 6). In 2018, a statistically significant negative correlation was found between ear weight and Fv/Fo (r = −0.97) as well as Fm/Fo (r = −0.97) at the 0 kg ha^−1^ level of N in the case of irrigated plots of Armagnac. In case of ear weight, further correlations were found in 2019 (Table 6). The mentioned parameter correlated well with Fv/Fm (r = −0.96) and Fv/Fo (r = −0.95) at a 160 kg ha^−1^ level of N in the case of irrigated plots of Armagnac and also with 0 kg ha^−1^ of N in the case of irrigated plots of Loupiac (r = 0.91). In the case of Armagnac, the Fv/Fm (r = −0.95), Fv/Fo (r = −0.97), and Fm/Fo (r = −0.97) parameters correlated with kernel weight at 0 kg ha^−1^ of N with irrigation. Results were similar in 2019 but then at a 160 kg ha^−1^ dose of N. In 2018, another correlation was found among fluorescence parameters and kernel weight at 80 and 160 kg ha^−1^ N levels in the Loupiac genotype (Table 6).

## 3. Discussion

The years of 2018 and 2019 were different from the viewpoint of maize production in Hungary. The amount of precipitation was appropriate in both years, but the distribution was unfavorable in 2018. Based on our statistical analysis, we found a difference between 2018 and 2019, but we did not find any interaction between the different vegetation periods and other factors, such as water, N supply, and genotypes. Micskei [28] found that most of the agronomical treatments have no effect on maize yield in drought years. In early phenological stages, the fertilizer levels did not influence any parameters, but there were differences between genotypes. Based on the measured parameters, the genotype is a main influence at this early growth stage. Less precipitation occurred in the April of 2019 than in 2018, and the average temperature was lower. These reasons explain why the measured parameters decreased more in 2019 than in 2018. Moussa and Abdel-Aziz [29] found differences in drought tolerance between genotypes based on physiological parameters in an early phenophase. 

At the VT phenophase, significantly higher values were found at an 80 kg ha^−1^ level of N in 2018, and the effect of irrigation was also remarkable in the actual photochemical efficiency. In 2019, the highest values were experienced at 160 kg N ha^−1^. The observations of Moser [30] were similar. According to them, the highest grain yield was achieved at 80 kg ha^−1^ of N under drought, whereas 160 kg N ha^−1^ resulted in the highest yield under well-watered conditions. N and water supply both affect the kernel number in maize [31]. Drought-tolerant genotypes are often tolerant to N deficiency [32]. Efeoğlu [33] declared that the chlorophyll fluorescence induction method and the measured parameters give reliable information about drought stress and the drought resistance of genotypes. The parameters measured by us indicate significant differences between genotypes, irrigation, and fertilizer levels. Significant interaction was found between irrigation and fertilizer level. Both kernel weight and ear weight were affected by N level and irrigation. The results of Uhart and Andrade [34] as well as of Reddy [35] were similar. The optimum N rate may be much lower than that used. Our results suggested the 80 kg ha^−1^ N dose could be optimal for maize, but it depends on environmental factors, such as soil type and weather conditions.

## 4. Materials and Methods

### 4.1. Experimental Design

Small block field experiments were set up at the trial site (Látókép) of the University of Debrecen (Hajdúság loess plateau, 47° 30′ N, 21° 36′ E, 121 m elevation) in 2018 and 2019. Three different maize (*Zea mays* L) hybrids were sown: Armagnac, Fornad, Loupiac. Treatments at different (0, 80, and 160 kg ha^−1^) levels of N and two irrigations, irrigated (25 mm + 25 mm) and non-irrigated, were used. The repetition number of small blocks was four.

### 4.2. Soil and Meteorological Conditions

The soil type of the experimental site is a lowland calcareous chernozem. The main parameters of the soil were the same as described by Nagy [36]. The soil pH (H_2_O) was 6.58. The soluble element content of the 0–0.3 depth layer of soil was the following: NO_3_ + NO_2_ 8.04 mg kg^−1^; P_2_O_5_ 199 mg kg^−1^ K_2_O 451 mg kg^−1^; Na 332 mg kg^−1^; Mg 176 mg kg^−1^; SO_4_^2-^ 6.04 mg kg; Cu 5.79 mg kg^−1^; Zn 7.9 mg kg^−1^; and Mn 262 mg kg^−1^. The organic matter concentration was 3.54%, and the CaCO3 concentration was 0.2%. The precipitation and temperature data were recorded from the meteorological station of the trial field. To describe the severity, the Gaussen–Banouls xerothermal index was used [37]. It was given as the ratio between monthly precipitation (mm) and the monthly average temperature multiplied by two (°C). According to the creators of this equation, if the value of the Gaussen–Banouls xerothermal index is lower than 1, the mentioned month can be described as a drought month.

### 4.3. Methods of Measurements

The parameters of in vivo chlorophyll fluorescence were detected with a PAM 2100 (Walz, Germany) modulated light fluorometer as described by Schreiber [38]. Samples were dark-adapted for 20 min. After a dark adaptation, the initial fluorescence (F_0_) was excited by a weak light (0.1 μmolm^−2^s^−1^). The maximal fluorescence (F_m_) was induced by a white saturating flash (8000 μmolm^−2^s^−1^) (fast phase of chlorophyll fluorescence). The parameters of the fast fluorescence induction phase were investigated: F_o_: initial fluorescence, F_m_: maximal fluorescence, F_v_ = F_m_−F_o_: variable fluorescence, F_v_/F_m_: potential photochemical efficiency of PSII, F_v_/F_o_: ratio of the variable and initial fluorescence parameters, F_m_/F_o_: ratio of the maximal and initial fluorescence parameters. The actual photochemical efficiency of PSII (ΔF/F_m_’ = (F_m_’−F_t_)/F_m_’) was measured in a light-acclimated condition under natural light. In vivo chlorophyll fluorescence measurements were carried out in three phenological phases (8 leaf (V8), tasseling (VT), silking (R1)) [39]. Measurements were made on the new fully developed leaves at the V8 stage and on the ear leaves at the VT and R1 stages. Total ear weight and kernel weight of individual ears were also determined and expressed in grams (g) at physiological maturity. The kernel/cob ratio was also calculated from the weight data. The weights were reported after drying at 65 °C to constant weight.

### 4.4. Data Management and Statistical Analysis

We used a completely randomized design with three N treatments, two irrigations, and three genotypes with four replications. For data management and statistical analysis, Microsoft Office Excel 2016 and SigmaPlot for Windows Version 12.0 were used. For the analysis, the methods of three way-ANOVA and Pearson Correlation were used. For all pairwise multiple comparisons, Duncan’s tests were used.

## 5. Conclusions

The optimal fertilizer dose depends on the water supply and temperature. Under dry conditions, 80 kg ha^−1^ is optimal for maize, while in optimal weather conditions, 160 kg ha^−1^ is optimal. Interaction between irrigation x N x genotype was found only at the VT stage. The parameters were less influenced by genotypes. The chlorophyll fluorescence induction method can provide reliable information during drought stress research. Lower N nutrition may be sufficient mainly under irrigated conditions, and in vivo chlorophyll fluorescence parameters are appropriate for detecting the effect of environmental factors in different growth stages.

Kernel weight and ear weight were affected by N dose and irrigation, but the kernel/cob ratio mostly depended on genotypes. According to the results of the correlation analysis, the chlorophyll fluorescence parameters correlated with ear parameters in just a few cases. Although the chlorophyll fluorescence parameters are widely applicable in stress research, the usability in yield estimation is limited.

## Figures and Tables

**Table 1:** Changes of Gaussen–Bagnouls xerothermal index from Jan 1 to Aug 31 in 2018 and 2019.

	January	February	March	April	May	June	July	August
2018	7.0	443.3	11.2	1.1	1.5	1.4	1.0	2.0
2019	−5.4	1.4	0.7	1.3	2.9	0.7	2.4	0.3

**Table 2:** Results of the three-way ANOVA performed on the Fo, Fm, Fv, Fv/Fm, Fv/Fo, and Fm/Fo parameters measured at the V8 and R1 stages of maize in different crop years (2018, 2019); changes are due to nitrogen supply (0, 80, 160 kg ha^−1^), irrigation (irrigated, non-irrigated), and genotype (Armagnac, Fornad, Loupiac) * *p* ≤ 0.05 (*n* = 4).

		2018							2019						
		Fo	Fm	Fv	Fv/Fm	Fv/Fo	Fm/Fo	Yield	Fo	Fm	Fv	Fv/Fm	Fv/Fo	Fm/Fo	Yield
V8	Genotype	n.s.	n.s.	*	n.s.	n.s.	n.s.	−	*	*	n.s.	*	n.s.	n.s.	−
	Nitrogen X Genotype	n.s.	n.s.	n.s.	n.s.	n.s.	n.s.	−	*	n.s.	n.s.	n.s.	n.s.	n.s.	−
R1	Genotype	n.s.	*	*	n.s.	n.s.	n.s.	n.s.	n.s.	n.s.	n.s.	n.s.	n.s.	n.s.	n.s.
	Nitrogen X Irrigation	*	n.s.	n.s.	n.s.	n.s.	n.s.	n.s.	n.s.	n.s.	n.s.	*	*	*	n.s.

**Table 3:** Changes of the Fo, Fm, Fv, Fv/Fm, Fv/Fo, Fm/Fo, and yield parameters at different levels of N (0, 80, 160 kg ha^−1^), genotypes (Armagnac, Fornad and Loupiac), and irrigation treatments (irrigated, non-irrigated) in 2018 (*n* = 4 ± s.e.).

		Armagnac			Fornad			Loupiac		
		0 kg ha^−1^	80 kg ha^−1^	160 kg ha^−1^	0 kg ha^−1^	80 kg ha^−1^	160 kg ha^−1^	0 kg ha^−1^	80 kg ha^−1^	160 kg ha^−1^
Irrigated	Fo	0.21 ± 0.02	0.21 ± 0.02	0.21 ± 0.01	0.19 ± 0.01	0.2 ± 0.01	0.21 ± 0.01	0.21 ± 0.01	0.22 ± 0.02	0.19 ± 0.03
	Fm	1.13 ± 0.12	1.02 ± 0.11	1.09 ± 0.13	0.86 ± 0.13	1.03 ± 0.14	1.08 ± 0.09	0.95 ± 0.16	1.15 ± 0.19	1.0 ± 0.16
	Fv	0.91 ± 0.1	0.81 ± 0.09	0.88 ± 0.12	0.67 ± 0.12	0.83 ± 0.13	0.87 ± 0.09	0.74 ± 0.15	0.93 ± 0.18	0.81 ± 0.14
	Fv/Fm	0.81 ± 0.01	0.79 ± 0.01	0.81 ± 0.02	0.78 ± 0.03	0.8 ± 0.01	0.81 ± 0.02	0.78 ± 0.02	0.81 ± 0.02	0.81 ± 0.03
	Fv/Fo	4.28 ± 0.34	3.85 ± 0.2	4.19 ± 0.43	3.64 ± 0.66	4.02 ± 0.39	4.27 ± 0.63	3.55 ± 0.52	4.19 ± 0.59	4.23 ± 0.65
	Fm/Fo	5.28 ± 0.34	4.85 ± 0.2	5.19 ± 0.44	4.64 ± 0.66	5.02 ± 0.39	5.27 ± 0.63	4.55 ± 0.52	5.19 ± 0.59	5.23 ± 0.65
	Yield	0.46 ± 0.11	0.46 ± 0.11	0.47 ± 0.1	0.42 ± 0.13	0.42 ± 0.09	0.44 ± 0.07	0.44 ± 0.06	0.4 ± 0.14	0.49 ± 0.09
Non-irrigated	Fo	0.22 ± 0.01	0.21 ± 0.02	0.21 ± 0.01	0.22 ± 0.02	0.2 ± 0.01	0.21 ± 0.01	0.21 ± 0.03	0.2 ± 0.01	0.21 ± 0.01
	Fm	1.2 ± 0.16	1.14 ± 0.09	1.08 ± 0.13	1.11 ± 0.12	1.04 ± 0.15	1.11 ± 0.07	1.08 ± 0.17	1.04 ± 0.01	1.04 ± 0.07
	Fv	0.98 ± 0.16	0.93 ± 0.08	0.87 ± 0.13	0.89 ± 0.1	0.84 ± 0.15	0.9 ± 0.06	0.86 ± 0.15	0.84 ± 0.07	0.84 ± 0.01
	Fv/Fm	0.81 ± 0.03	0.82 ± 0.01	0.8 ± 0.02	0.8 ± 0.01	0.8 ± 0.03	0.81 ± 0.01	0.8 ± 0.02	0.81 ± 0.01	0.8 ± 0.02
	Fv/Fo	4.42 ± 0.7	4.5 ± 0.28	4.12 ± 0.56	4.01 ± 0.26	4.17 ± 0.73	4.24 ± 0.14	4.05 ± 0.47	4.24 ± 0.28	4.06 ± 0.46
	Fm/Fo	5.42 ± 0.71	5.5 ± 0.28	5.12 ± 0.56	5.01 ± 0.26	5.17 ± 0.73	5.24 ± 0.14	5.05 ± 0.47	5.25 ± 0.28	5.06 ± 0.45
	Yield	0.5 ± 0.1	0.54 ± 0.04	0.43 ± 0.12	0.38 ± 0.09	0.39 ± 0.09	0.39 ± 0.14	0.48 ± 0.11	0.47 ± 0.11	0.47 ± 0.11

**Table 4:** Changes of the Fo, Fm, Fv, Fv/Fm, Fv/Fo, Fm/Fo, and yield parameters at different levels of N (0, 80, 160 kg ha^−1^), genotypes (Armagnac, Fornad, and Loupiac), and irrigation treatments (irrigated, non-irrigated) in 2019 (*n* = 4 ± s.e.).

		Armagnac			Fornad			Loupiac		
		0 kg ha^−1^	80 kg ha^−1^	160 kg ha^−1^	0 kg ha^−1^	80 kg ha^−1^	160 kg ha^−1^	0 kg ha^−1^	80 kg ha^−1^	160 kg ha^−1^
Irrigated	Fo	0.24 ± 0.01	0.24 ± 0.02	0.23 ± 0.01	0.23 ± 0.02	0.24 ± 0.03	0.24 ± 0.03	0.25 ± 0.01	0.24 ± 0.01	0.25 ± 0.02
	Fm	1.22 ± 0.08	1.26 ± 0.2	1.22 ± 0.14	1.15 ± 0.06	1.29 ± 0.18	1.23 ± 0.07	1.26 ± 0.05	1.09 ± 0.11	1.33 ± 0.12
	Fv	0.98 ± 0.09	1.02 ± 0.19	0.98 ± 0.14	0.91 ± 0.05	1.05 ± 0.16	0.99 ± 0.06	1.01 ± 0.06	0.85 ± 0. 11	1.08 ± 0.11
	Fv/Fm	0.81 ± 0.02	0.81 ± 0.03	0.81 ± 0.03	0.8 ± 0.02	0.81 ± 0.02	0.81 ± 0.02	0.8 ± 0.02	0.78 ± 0.03	0.81 ± 0.01
	Fv/Fo	4.15 ± 0.42	4.25 ± 0.71	4.27 ± 0.66	4.02 ± 0.35	4.41 ± 0.53	4.24 ± 0.46	3.91 ± 0.45	3.61 ± 0.53	4.38 ± 0.24
	Fm/Fo	5.15 ± 0.42	5.25 ± 0.71	5.27 ± 0.66	4.91 ± 0.35	5.41 ± 0.53	5.24 ± 0.46	5.02 ± 0.45	4.61 ± 0.53	5.39 ± 0.24
	Yield	0.68 ± 0.06	0.67 ± 0.08	0.59 ± 0.17	0.63 ± 0.08	0.69 ± 0.09	0.64 ± 0.12	0.62 ± 0.16	0.46 ± 0.14	0.62 ± 0.08
Non-irrigated	Fo	0.26 ± 0.02	0.23 ± 0.01	0.25 ± 0.01	0.24 ± 0.01	0.25 ± 0.02	0.23 ± 0.03	0.25 ± 0.01	0.25 ± 0.02	0.24 ± 0.02
	Fm	1.25 ± 0.24	1.26 ± 0.17	1.08 ± 0.19	1.19 ± 0.15	1.3 ± 0.1	1.19 ± 0.12	1.13 ± 0.15	1.31 ± 0.19	1.08 ± 0.14
	Fv	1.0 ± 0.22	1.03 ± 0.16	0.84 ± 0.19	0.95 ± 0.14	1.06 ± 0.08	0.95 ± 0.09	0.88 ± 0.15	1.05 ± 0.17	0.84 ± 0.12
	Fv/Fm	0.79 ± 0.03	0.82 ± 0.02	0.77 ± 0.05	0.8 ± 0.02	0.81 ± 0.01	0.8 ± 0.01	0.78 ± 0.04	0.81 ± 0.01	0.78 ± 0.02
	Fv/Fo	3.89 ± 0.71	4.47 ± 0.58	3.44 ± 0.86	4 ± 0.54	4.31 ± 0.26	4.07 ± 0.11	3.55 ± 0.7	4.17 ± 0.38	3.47 ± 0.31
	Fm/Fo	4.89 ± 0.71	5.47 ± 0.59	4.44 ± 0.86	5 ± 0.53	5.31 ± 0.26	5.07 ± 0.11	4.54 ± 0.7	5.17 ± 0.38	4.46 ± 0.31
	Yield	0.66 ± 0.07	0.53 ± 0.14	0.62 ± 0.09	0.49 ± 0.33	0.55 ± 0.27	0.34 ± 0.39	0.56 ± 0.12	0.71 ± 0.03	0.58 ± 0.11

**Table 5:** Changes in weight of ear (g), kernel (g), and kernel/cob ratio at different levels of N (0, 80, 160 kg ha-1) in different crop years (2018, 2019), genotypes (Armagnac, Fornad, and Loupiac), and irrigation treatments (irrigated, non-irrigated) (*n* = 4 ± s.e.).

			Armagnac			Fornad			Loupiac		
			0 kg ha^−1^	80 kg ha^−1^	160 kg ha^−1^	0 kg ha^−1^	80 kg ha^−1^	160 kg ha^−1^	0 kg ha^−1^	80 kg ha^−1^	160 kg ha^−1^
Irrigated	2018	Ear (g)	206.59 ± 46.93	219.7 ± 32.83	231.36 ± 10.16	142.61 ± 26.5	228.71 ± 16.55	211.67 ± 28.08	178.05 ± 59.74	214.63 ± 21.62	216.95 ± 20.72
		Kernel (g)	180.77 ± 41.15	195.65 ± 28.31	205.39 ± 8.86	127.68 ± 23.86	207.56 ± 15.05	191.68 ± 23.84	159.66 ± 53.46	193.77 ± 19.91	191.02 ± 25.63
		Ker/cob ratio	7.01 ± 0.25	8.24 ± 0.81	7.92 ± 0.25	8.63 ± 1.08	9.82 ± 0.27	9.73 ± 0.88	8.69 ± 0.73	9.4 ± 1.28	7.8 ± 2.28
	2019	Ear (g)	178.3 ± 53.25	180.42 ± 41.95	226.22 ± 19.41	163.33 ± 38.1	173.17 ± 12.76	199.41 ± 28.99	167.64 ± 43.57	208.58 ± 14.87	225.22 ± 10.28
		Kernel (g)	156.19 ± 48.93	158.24 ± 38.24	199.77 ± 16.57	143.57 ± 34.26	156.01 ± 11.5	180.44 ± 25.34	149.49 ± 38.12	187.37 ± 12.87	202.31 ± 8.27
		Ker/cob ratio	6.96 ± 1.11	7.07 ± 0.63	7.57 ± 0.33	7.33 ± 0.98	9.12 ± 0.64	9.59 ± 0.53	8.31 ± 0.38	8.91 ± 0.86	8.92 ± 0.93
Non- irrigated	2018	Ear (g)t	182.87 ± 56.35	201.86 ± 30.42	213.61 ± 32.62	185.86 ± 30.18	192.02 ± 15.69	173.58 ± 13	160.54 ± 40.72	221.52 ± 42.71	201.67 ± 16.42
		Kernel (g)	159.99 ± 50.07	177.69 ± 26.44	188.51 ± 27.92	168.88 ± 28.38	174.33 ± 14.19	157.63 ± 11.09	141.81 ± 38.81	198.31 ± 37.87	177.41 ± 17.13
		Ker/cob ratio	6.97 ± 0.51	7.37 ± 0.19	7.58 ± 0.68	9.9 ± 0.62	9.95 ± 1.14	9.96 ± 0.84	7.74 ± 1.86	8.6 ± 0.57	7.45 ± 1.58
	2019	Ear (g)	168.77 ± 47.29	173.82 ± 47.24	184.48 ± 18.73	137.36 ± 44.47	202.26 ± 32.35	198.29 ± 19.63	143.51 ± 28.47	172.89 ± 23.44	187.1 ± 28.43
		Kernel (g)	148.33 ± 39.95	153.8 ± 43.19	162.25 ± 15.42	123.97 ± 40.41	180.71 ± 29.64	178.93 ± 17.09	127.53 ± 25.52	155.18 ± 21.31	166.86 ± 25.69
		Ker/cob ratio	7.62 ± 1.76	7.61 ± 0.77	7.34 ± 0.37	9.4 ± 1.62	8.47 ± 1.2	9.28 ± 0.48	7.97 ± 0.2	8.81 ± 0.94	8.27 ± 0.83

**Table 6:** Changes of the correlation coefficients (r) between ear parameters of maize (ear weight, kernel weight) and chlorophyll fluorescence parameters (Fv/Fm, Fv/Fo, Fm/Fo) at different levels of N (0, 80, 160 kg ha^−1^) in different crop years (2018, 2019), genotypes (Armagnac, Fornad and Loupiac), and irrigation treatments (irrigated, non-irrigated). Significant differences in grey (*p* ≤ 0.05) and dark grey (*p* ≤ 0.01).

				0 kg ha^−1^			80 kg ha^−1^			160 kg ha^−1^		
				Fv/Fm	Fv/Fo	Fm/Fo	Fv/Fm	Fv/Fo	Fm/Fo	Fv/Fm	Fv/Fo	Fm/Fo
Ear weight	Armagnac	2018	Irrigated	−0.95	−0.97	−0.97	0.85	0.83	0.83	0.49	0.8	0.48
			Non-irrigated	−0.90	−0.90	−0.90	−0.63	−0.65	−0.66	−0.54	−0.54	−0.54
		2019	Irrigated	0.66	0.69	0.70	0.37	0.39	0.40	0.96	0.95	0.95
			Non-irrigated	−0.69	−0.67	−0.67	0.48	0.52	0.53	0.28	0.24	0.24
	Fornad	2018	Irrigated	0.90	0.88	0.88	−0.15	−0.15	−0.15	0.82	0.84	0.84
			Non-irrigated	0.82	0.86	0.86	−0.02	−0.01	−0.01	−0.05	0.00	0.01
		2019	Irrigated	0.07	0.02	0.01	0.90	−0.91	−0.91	0.44	0.43	0.43
			Non-irrigated	0.82	0.81	0.81	0.34	0.34	0.33	0.65	0.63	0.62
	Loupiac	2018	Irrigated	−0.26	−0.22	−0.22	−1.00	−0.99	−0.99	0.93	0.95	0.94
			Non-irrigated	0.53	0.55	0.55	0.07	0.10	0.10	−0.63	−0.66	−0.66
		2019	Irrigated	0.93	0.91	0.91	0.63	0.59	0.59	−0.59	−0.55	−0.55
			Non-irrigated	−0.49	−0.49	−0.50	0.89	0.88	0.88	0.83	0.85	0.85
Kernel weight	Armagnac	2018	Irrigated	−0.95	−0.97	−0.97	0.84	0.82	0.82	0.42	0.41	0.41
			Non-irrigated	−0.89	−0.89	−0.89	−0.62	−0.65	−0.65	−0.59	−0.60	−0.59
		2019	Irrigated	0.67	0.70	0.71	0.35	0.38	0.38	0.97	0.97	0.96
			Non-irrigated	−0.66	−0.64	−0.64	0.50	0.54	0.55	0.28	0.24	0.23
	Fornad	2018	Irrigated	0.91	0.89	0.89	−0.11	−0.11	−0.12	0.82	0.84	0.60
			Non−irrigated	0.82	0.85	0.85	0.10	0.12	0.12	0.01	0.05	0.06
		2019	Irrigated	0.14	0.09	0.07	−0.87	−0.88	−0.88	0.43	0.43	0.42
			Non−irrigated	0.86	0.85	0.84	0.79	0.42	0.41	0.63	0.61	0.60
	Loupiac	2018	Irrigated	−0.23	−0.20	−0.20	−1.00	−1.00	−1.00	0.98	0.97	−0.39
			Non−irrigated	0.58	0.61	0.61	0.06	0.08	0.08	−0.58	−0.63	−0.63
		2019	Irrigated	0.93	0.91	0.90	0.62	0.59	0.59	−0.66	−0.63	−0.62
			Non−irrigated	−0.50	−0.50	−0.50	−0.05	0.89	0.89	0.83	0.84	0.85
